# Trait‐based ecology of terrestrial arthropods

**DOI:** 10.1111/brv.12488

**Published:** 2018-12-13

**Authors:** Mark K. L. Wong, Benoit Guénard, Owen T. Lewis

**Affiliations:** ^1^ Department of Zoology University of Oxford Oxford, OX1 3PS U.K.; ^2^ School of Biological Sciences The University of Hong Kong, Kadoorie Biological Sciences Building Hong Kong SAR China

**Keywords:** functional trait, functional diversity, community assembly, ecosystem function, insect, invertebrate, ant, bee, beetle, butterfly, spider, review

## Abstract

In focusing on how organisms' generalizable functional properties (traits) interact mechanistically with environments across spatial scales and levels of biological organization, trait‐based approaches provide a powerful framework for attaining synthesis, generality and prediction. Trait‐based research has considerably improved understanding of the assembly, structure and functioning of plant communities. Further advances in ecology may be achieved by exploring the trait–environment relationships of non‐sessile, heterotrophic organisms such as terrestrial arthropods, which are geographically ubiquitous, ecologically diverse, and often important functional components of ecosystems. Trait‐based studies and trait databases have recently been compiled for groups such as ants, bees, beetles, butterflies, spiders and many others; however, the explicit justification, conceptual framework, and primary‐evidence base for the burgeoning field of ‘terrestrial arthropod trait‐based ecology’ have not been well established. Consequently, there is some confusion over the scope and relevance of this field, as well as a tendency for studies to overlook important assumptions of the trait‐based approach. Here we aim to provide a broad and accessible overview of the trait‐based ecology of terrestrial arthropods. We first define and illustrate foundational concepts in trait‐based ecology with respect to terrestrial arthropods, and justify the application of trait‐based approaches to the study of their ecology. Next, we review studies in community ecology where trait‐based approaches have been used to elucidate how assembly processes for terrestrial arthropod communities are influenced by niche filtering along environmental gradients (e.g. climatic, structural, and land‐use gradients) and by abiotic and biotic disturbances (e.g. fire, floods, and biological invasions). We also review studies in ecosystem ecology where trait‐based approaches have been used to investigate biodiversity–ecosystem function relationships: how the functional diversity of arthropod communities relates to a host of ecosystem functions and services that they mediate, such as decomposition, pollination and predation. We then suggest how future work can address fundamental assumptions and limitations by investigating trait functionality and the effects of intraspecific variation, assessing the potential for sampling methods to bias the traits and trait values observed, and enhancing the quality and consolidation of trait information in databases. A roadmap to guide observational trait‐based studies is also presented. Lastly, we highlight new areas where trait‐based studies on terrestrial arthropods are well positioned to advance ecological understanding and application. These include examining the roles of competitive, non‐competitive and (multi‐)trophic interactions in shaping coexistence, and macro‐scaling trait–environment relationships to explain and predict patterns in biodiversity and ecosystem functions across space and time. We hope this review will spur and guide future applications of the trait‐based framework to advance ecological insights from the most diverse eukaryotic organisms on Earth.

## I. INTRODUCTION

Biodiversity is often described exclusively in terms of the distinct taxonomic entities (species) which it contains, and measured in terms of its taxonomic component, taxonomic diversity (e.g. species richness). Taxonomic approaches, however, offer limited insight into the evolutionary and mechanistic underpinnings of ecological phenomena; these have recently been studied using alternative approaches for describing biodiversity. For instance, phylogenetic approaches emphasize organisms' evolutionary affiliations, and measure the phylogenetic component of biodiversity, phylogenetic diversity (Cadotte *et al*., 2010). By contrast, functional trait‐based approaches (henceforth ‘trait‐based approaches’) emphasize the values of organisms' phenotypic traits, whose interactions with biotic and abiotic environments affect organism fitness – hence the term ‘functional’ (McGill *et al*., 2006; Violle *et al*., 2007). In measuring the diversity of traits and trait values (the values of traits at specific points along environmental gradients), trait‐based approaches measure the functional component of biodiversity, functional diversity (Díaz & Cabido, 2001; Petchey & Gaston, 2006; Violle *et al*., 2007).

Studies using trait‐based approaches to investigate ecological relationships have proliferated over the past decade (McGill, 2015). The rapid rise of ‘trait‐based ecology’ has been propelled by its promise of synthesis, generality and prediction (see Section II.2, and Shipley *et al*., 2016). Most progress has been witnessed in plant ecology (reviewed in Funk *et al*., 2017), where trait‐based approaches are now widely employed to investigate the processes underlying patterns of species coexistence (Cornwell & Ackerly, 2009; Kunstler *et al*., 2016) and biodiversity and ecosystem function (BEF) relationships (Cadotte, Carscadden & Mirotchnick, 2011; Faucon, Houben & Lambers, 2017). Extensive databases of plant traits (Kattge *et al*., 2011) are also available to ecologists.

Trait‐based approaches were introduced to plant ecology in a series of highly cited papers published during the first decade of the 21st century (e.g. Díaz & Cabido, 2001; Lavorel & Garnier, 2002; Díaz *et al*., 2004; McGill *et al*., 2006; Petchey & Gaston, 2006; Violle *et al*., 2007), but can be traced to earlier work (e.g. Weiher & Keddy, 1995a, 1995b). Soon, they were incorporated in research on microbes (Green, Bohannan & Whitaker, 2008; Krause *et al*., 2014), and animals including vertebrates (Luck *et al*., 2012), aquatic invertebrates (Poff *et al*., 2006), and terrestrial arthropods such as ants, bees, beetles, butterflies, and spiders (Pey *et al*., 2014; Moretti *et al*., 2017; Perović *et al*., 2018; Brousseau, Gravel & Handa, 2018a). Researchers working on these groups have recently established protocols for selecting and measuring traits (Fountain‐Jones, Baker & Jordan, 2015; Moretti *et al*., 2017), extensive databases consolidating trait information (Homburg *et al*., 2014; Parr *et al*., 2017), and guidelines for using trait‐based approaches to enhance biological control services in managed landscapes (Perović *et al*., 2018; Gardarin *et al*., 2018). Recently, Brousseau *et al*. (2018a) also reviewed trait‐based studies on terrestrial arthropods to identify the traits that were used, and how these related to the studied ecological filters (*sensu* Keddy, 1992). Evidently, considerable efforts are underway to navigate the technicalities – the ‘hows’ – of using trait‐based approaches in both empirical and applied studies on terrestrial arthropods. However, the explicit justification, conceptual framework, and primary evidence base for the burgeoning field of ‘terrestrial arthropod trait‐based ecology’ have not been well established. Consequently, there is some confusion over the scope and relevance of this field, as well as a tendency for studies to overlook important assumptions of the trait‐based approach (Didham, Leather & Basset, 2016).

Here we provide a broad and, we hope, accessible overview of trait‐based ecology as applied to terrestrial arthropods. We define and justify this field, summarize existing knowledge, suggest how future work can address current limitations, and highlight new areas of research that will have most impact. Specifically, our review addresses the following questions: what is a trait and what is trait‐based ecology? Why are trait‐based approaches relevant to the study of terrestrial arthropod ecology? What areas in ecology have been explored in current trait‐based studies on terrestrial arthropods? What are the assumptions and limitations of trait‐based studies, and how can these be addressed? What new areas in ecology should be explored in future trait‐based studies on terrestrial arthropods?

As trait‐based studies encompass multiple subdisciplines of ecology, and considering the spectacular diversity of terrestrial arthropods (estimated at 7 million species globally; Stork, 2017), it is impossible to cover all relevant material within this review. Although our discussion draws primarily from research on commonly studied groups (ants, bees, beetles, butterflies and spiders), the underlying framework as well as the opportunities and challenges of trait‐based research presented here should be relevant to similar work on the majority of terrestrial arthropods. We hope this review will establish a preliminary knowledge base for the exciting field of terrestrial arthropod trait‐based ecology, and guide future applications of the trait‐based framework to advance ecological insights from the most diverse eukaryotic organisms on Earth.

### (1) Defining a trait

Technical applications of trait‐based approaches vary among fields and are shaped by new developments, but studies of both plants and animals agree on the general properties of traits (by ‘traits’ we mean functional traits). These are twofold: (*i*) traits are phenotypic entities that are strictly measured on individual organisms; and (*ii*) traits are functional, in the sense that their interactions with biotic and abiotic environments affect performance, and consequently organism fitness (McGill *et al*., 2006; Violle *et al*., 2007) – we term this ‘fitness‐functionality’. For the many trait‐based studies investigating BEF relationships, traits should also be functional in the sense that they impact or regulate higher‐level ecological processes and patterns (Mlambo, 2014; Schmitz *et al*., 2015) – we term this ‘ecosystem‐functionality’. Importantly, and as highlighted by previous authors (Pey *et al*., 2014; Middleton‐Welling *et al*., 2018), several studies incorrectly labelled as ‘traits’ environmental properties associated with species occurrences. Some examples we encountered include ‘habitat openness’ (Eskildsen *et al*., 2015) and ‘moisture preference’ (Pakeman & Stockan, 2014). While the former is a measure of vegetation, the latter is based on occurrence distributions of multiple individuals of a species along an environmental gradient. Since these properties are not measured on individual organisms, they should be distinguished from traits. They might more appropriately be termed ‘ecological preferences’ (Pey *et al*., 2014).

To facilitate comparisons across studies, traits are often broadly categorized according to the particular aspects of phenotype that they describe. Moretti *et al*. (2017) proposed five categories of traits for terrestrial invertebrates: morphology (body size, eye number, etc.), feeding (ingestion rate, biting force, etc.), life history (ontogeny, clutch size, etc.), physiology (resting metabolic rate, relative growth rate, etc.), and behaviour (activity time, sociality, etc.). An extensive list of the traits across these five categories as used in existing trait‐based studies on terrestrial arthropods was recently made available (see Table S2 in Brousseau *et al*., 2018a). Depending on the specific ecological question at hand, however, individual studies may further distinguish traits based on the impacts of their interactions. Two common examples – which are not mutually exclusive – are the response–effect paradigm (Lavorel & Garnier, 2002; Suding *et al*., 2008), and the performance paradigm (Violle *et al*., 2007).

The response–effect paradigm (Lavorel & Garnier, 2002; Suding *et al*., 2008) considers the impacts of trait interactions with the environment. Here, traits may be identified as ‘response traits’ – the attributes of which vary in their responses to environmental conditions (e.g. in lepidopterans, larval diet specialization determines responses to changes in habitat composition; Aguirre‐Gutiérrez *et al*., 2016); or ‘effect traits’ – the attributes of which vary in their effects on ecosystem properties (e.g. in dung beetles, body size affects the efficiency of dung removal and seed burial; Slade *et al*., 2007). In general, effect traits influence the performance of ecosystem functions, whereas response traits influence their resilience (Lavorel & Garnier, 2002; Violle *et al*., 2007; Wright, Ames & Mitchell, 2016). Response traits and effect traits can be interlinked; for instance, body sizes of bees and dung beetles are informative as both response traits and effect traits (Larsen, Williams & Kremen, 2005). Studies on plants have established links between response and effect traits that facilitate predictions about the effects of environmental changes on community dynamics (responses) and the ecosystem functions mediated by these communities (effects) (Suding & Goldstein, 2008; Fortunel *et al*., 2009); similar work has emerged in studies on terrestrial arthropods (see Section II.2).

The hierarchical performance paradigm (Violle *et al*., 2007) identifies traits that essentially describe individual performance in growth, reproduction and survival – the three components of individual fitness (Arnold, 1983), and distinguishes these ‘performance traits’ from other functional traits that are ‘lower’ on the performance hierarchy, which only impact fitness indirectly through their influence on growth, reproduction and survival. For instance, the three plant performance traits – vegetative biomass, reproductive output, and plant survival – are distinguished from other functional traits such as leaf morphology and wood density (Violle *et al*., 2007). To the best of our knowledge, the performance paradigm has not been explicitly incorporated in trait‐based studies on terrestrial arthropods; often, data on traits describing performance (e.g. clutch size) are combined with data on other functional traits that indirectly affect performance (e.g. wingspan). However, employing the performance paradigm to link lower traits to performance (and fitness) may contribute to addressing the fundamental assumption of fitness‐functionality in observational studies where this is often a challenge (see Section III.1*b*). The performance paradigm may also be relevant to future trait‐based studies investigating competition and coexistence, where it may be useful to distinguish performance traits that potentially provide competitive advantages, or which directly impact growth rates, from other traits that may otherwise contribute to stabilization (see Section IV.2).

### (2) What is trait‐based ecology?

Trait‐based ecology is the study of how the generalizable, functional properties of individual organisms – their traits – interact with abiotic and biotic environments across different levels of biological organization. Here, it is organisms' traits – and not their species identities – that are viewed as the common currency across biological organizational levels and taxonomic groups (Violle *et al*., 2014). Trait‐based approaches facilitate the testing of hypotheses to reveal the ecological mechanisms which determine how individual traits interact with abiotic and biotic environments *via* their responses and effects. This makes it possible to aggregate and integrate different traits to explain structure and functioning mechanistically across different scales of organization (i.e. populations, communities, ecosystems, biomes, and beyond) (Violle *et al*., 2014). In this way, trait‐based ecology facilitates the synthesis of generalizable (i.e. comparable; independent of geographical location or taxonomic assemblage) and predictive (i.e. based on knowledge of mechanisms) explanations for multiple ecological phenomena. Together with the increasing availability of trait values in the literature – epitomized in massive trait databases (e.g. Kattge *et al*., 2011) – this promise of generality, synthesis, and predictive ability accounts for the burgeoning prominence of trait‐based ecological research (Shipley *et al*., 2016).

**Figure 1:**
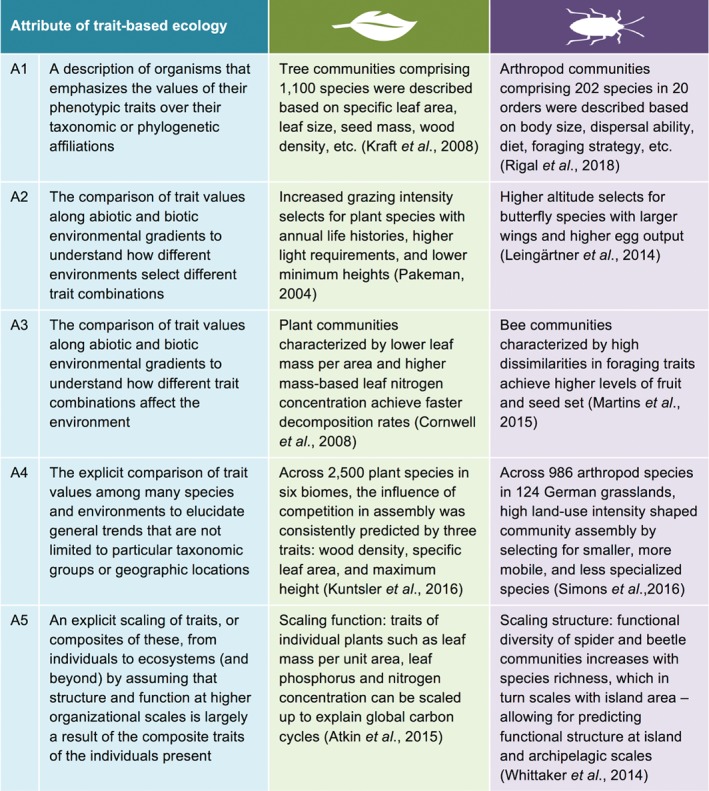
Attributes of trait‐based ecology (modified after Shipley *et al*., 2016). Five defining attributes of trait‐based ecology (A1–5) are listed, with examples from studies on plants and terrestrial arthropods. These attributes distinguish studies using trait‐based approaches from those using other approaches (e.g. taxonomy and phylogenetics). Not all trait‐based studies display all attributes, but at minimum they should display A1; thereafter, depending on the specific ecological question, studies may display one or a combination of the other attributes (A2–5). Note that trait‐based research is not constrained to a particular organizational scale or set of ecological phenomena; for instance, studies displaying A2 may investigate community ecology, studies displaying A3 may investigate ecosystem ecology, and studies displaying A5 may investigate macroecology.

As ecology is the study of organisms and their interactions, and as all organisms have traits, there is unsurprisingly some confusion over the types of studies that constitute trait‐based ecology. Clarification was recently provided by Shipley *et al*. (2016): trait‐based ecology is not defined by the ecological phenomena that it studies, or the organizational scale at which it is studied (as for subdisciplines such as ‘population ecology’, ‘community ecology’ and ‘ecosystem ecology’), but rather by the *way* that it studies them. In Fig. 1 we list several defining attributes of trait‐based studies (after Shipley *et al*., 2016) and provide examples for plants as well as terrestrial arthropods.

It should be emphasized that trait‐based approaches are not (and should not be) constrained to examining one particular ecological theory or question; the predictive value of trait‐based approaches essentially stems from their versatile potential to test different ecological theories empirically, and reveal mechanisms across different organizational scales. Much of the existing research on terrestrial arthropods has applied trait‐based approaches to investigate community assembly within the context of ecological filters (Keddy, 1992; Shipley, Vile & Garnier, 2006; see examples in Section II.1, and Brousseau *et al*., 2018a); although only a minority (19%) of these clearly postulated hypotheses linking traits, their functions, and the studied environmental filters (Brousseau *et al*., 2018a). Other such studies are limited to describing correlations between trait values and environmental properties (Brousseau *et al*., 2018a). While purely descriptive studies on trait–environment relationships do contribute some information about ecological structure, the dearth of theory‐driven investigations represents lost opportunities for elucidating specific ecological mechanisms underlying observed patterns, effectively undermining the predictive value of trait‐based research. In addition to the current scope of trait‐based research on terrestrial arthropods, more theory‐driven applications are possible (some examples are given in Section IV) and further such work will be required to facilitate synthesis and generality.

Trait‐based studies often measure functional diversity – the diversity of traits and trait values – so as to describe the diversity of forms and functions within a particular study unit. Functional diversity is comprised of three main aspects: functional richness, the volume of multidimensional trait space that is occupied; functional evenness, the distribution of abundance in multidimensional trait space; and functional divergence, the degree to which the distribution of abundance in multidimensional trait space maximizes differences in trait values (Mason *et al*., 2005; Villéger, Mason & Mouillot, 2008). These aspects of functional diversity can be measured using a variety of metrics such as functional richness (FRic), Rao's quadratic entropy (*Q*), functional divergence (FDiv), and functional evenness (FEve); see Mouchet *et al*. (2010) for a review and discussion on the use of different functional diversity metrics.

### (3) Why a trait‐based ecology of terrestrial arthropods?

In addition to the notable contributions of trait‐based studies on plants, trait‐based research on other taxa has considerable potential to improve ecological theory and practice. We suggest that terrestrial arthropods represent an ideal group for such work because their taxonomic and ecological diversity is unmatched. Most eukaryotic species on Earth are terrestrial arthropods (Zhang, 2013; Stork, 2017); they are ubiquitous throughout the terrestrial biosphere, and the biomass of groups such as ants and termites commonly exceeds that of larger vertebrate animals (Fittkau & Klinge, 1973). Hence, trait‐based research on terrestrial arthropods can contribute generalizable, mechanistic explanations for the processes generating and maintaining the diversity of non‐sessile, heterotrophic organisms across a variety of habitats, environmental gradients, and spatial scales. Trait‐based research on terrestrial arthropods will also advance understanding and prediction of numerous ecosystem processes and services (Losey & Vaughan, 2006) that are still poorly understood, and for which terrestrial arthropods are major contributors in their diverse roles as herbivores, fungivores, granivores, detritivores, predators, and parasites. For instance, as ecosystem engineers, ants and termites extensively modify soil properties, disproportionately affecting the fitness of other organisms (Lavelle *et al*., 2006); spiders consume insects at globally significant levels (Nyffeller & Birkhofer, 2017), in turn altering plant diversity and productivity (Schmitz, 2003); and many Hymenoptera, Diptera and Lepidoptera are important pollinators (Potts *et al*., 2010).

To facilitate trait‐based research on terrestrial arthropods, there is an abundance of information in the literature and extensive physical and digital collections (Short, Dikow & Moreau, 2018). The trait‐based approach is also an avenue for overcoming the many taxonomic impediments that have long plagued ecological research on terrestrial arthropods. These impediments include: the great majority of species remaining undescribed – and even less understood ecologically (Cardoso *et al*., 2011; Hortal *et al*., 2015); the sheer diversity and abundance of cryptic species, even in common, functionally important taxa (Molbo *et al*., 2003; Murray *et al*., 2008); and poor standards of taxonomic treatment in most ecological studies, undermining validation and reproducibility (Packer *et al*., 2018). To this end, we envision that ecological studies incorporating a focus on traits and their interactions (and thus not being constrained to taxonomic affiliations) will cut to the mechanistic bases of ecological relationships; however taxonomic excellence is still crucial for macroecological trait‐based research integrating data from multiple studies, and for the development and maintenance of trait databases (see Section III.4). Together, trait‐based studies across the diversity of terrestrial arthropod taxa and habitats could provide a broad, comparative framework (with traits as standard properties) for investigating fundamental and applied ecological questions. This is particularly important for expediting understanding of the ecology and functioning of threatened systems such as tropical forests, which support – and are in turn supported by – high levels of arthropod diversity (Basset *et al*., 2012).

### (4) How novel are trait‐based studies of terrestrial arthropods?

It should be noted that a focus on terrestrial arthropods' traits and their relationships with environmental gradients is not entirely new. For instance, many earlier studies on Bergmann's rule in insects (e.g. Park, 1949; Masaki, 1967; Hawkins & Lawton, 1995) investigated trait–environment relationships, although these may not have been stated explicitly (in earlier studies traits were often referred to as ‘characters’). Nevertheless, the latest wave of trait‐based studies on terrestrial arthropods – those incorporating the trait‐based framework outlined in plant studies from the first decade of the 21st century (examples cited above) – do represent a distinct shift from the previous era where terrestrial arthropod functional ecology was predominantly investigated *via* a functional group approach akin to that used in plants (e.g. Tilman *et al*., 1997). This involved assigning species to different functional groups *a priori*, based on their observed or assumed biotic and abiotic interactions (functions); the number of groups within a particular scale of biological organization determined its functional diversity. Examples of widely used functional groupings include those for ants and termites, based on taxonomic relationships and diet specialization (Andersen, 1995; Donovan *et al*., 2001); and dung beetles, based on method of dung removal (Doube, 1990). However, the functional group approach is problematic for a number of reasons (Villéger *et al*., 2008). For instance, groupings impose a discrete structure on functional differences that are usually continuous, resulting in a loss of information (Gitay & Noble, 1997); relationships observed are dependent on the specific functional grouping selected from an often wide variety of options (Wright *et al*., 2006); functional groups fail at accounting for the effects of abundance (Díaz & Cabido, 2001); and functional groups fail on their promise of generality because they were developed based on few assemblages in specific locations (Bourguignon *et al*., 2011). Hence, in addition to the relative success of trait‐based plant ecology, significant limitations of the functional group approach lent impetus to the current ascent of terrestrial arthropod trait‐based ecology.

## II. CURRENT TRAIT‐BASED STUDIES ON TERRESTRIAL ARTHROPODS

In this section we review knowledge in two broad and related areas of ecology that have received the most attention from existing trait‐based studies on terrestrial arthropods. The first area is the study of community assembly based on patterns in community functional structure. Most such studies investigate deterministic assembly mechanisms, in particular the influence of niche filtering along environmental gradients, while others investigate how abiotic and biotic disturbances influence assembly processes and shape community functional structure. The second area investigates how community functional structure affects the performance of ecosystem functions, that is, BEF relationships.

### (1) Elucidating community assembly

‘Community’ refers to a set of species with shared ecological characteristics that coexist in the same area (Chesson, 2000). Community assembly, the processes by which species from a regional pool colonize and coexist in the same area (HilleRisLambers *et al*., 2012), may occur deterministically through niche‐based mechanisms, as well as stochastically through niche‐independent processes, such as dispersal, colonization and extinction (Chase & Myers, 2011). Here, the niche comprises both a species' responses to and its effects on the abiotic and biotic environmental properties required for its survival and reproduction (Chase & Leibold, 2003). Since species' interactions with abiotic and biotic environments occur through their traits, the particular composition and distribution of traits and trait values among species in a community (i.e. the community's functional structure or functional diversity) may be interpreted as the pattern of niche occupation by species in that community (McGill *et al*., 2006). Community functional structure provides limited insight into stochastic processes because these are niche independent (Funk *et al*., 2017); however, if deterministic mechanisms have influenced community assembly, these should generate non‐random community functional structure – comprising only traits that successfully exploit available niches. For instance, the strength of deterministic mechanisms in the assembly of ant communities in rubber plantations (Liu *et al*., 2016) was inferred from patterns of functional diversity significantly deviating from null‐modelled expectations of random structure. By contrast, random‐like patterns in morphological traits of ant communities in Brazilian Atlantic forests suggested that deterministic mechanisms (relating to the niches represented by those traits) had little influence on their assembly (Silva & Brandão, 2014).

In addition to establishing the overall deterministic nature of the assembly process, trait‐based approaches can be used to reveal how specific niche‐based mechanisms operate in community assembly. Thus far, trait‐based studies on terrestrial arthropods have predominantly investigated how the mechanism of niche filtering influences community assembly along various environmental gradients (see Section II.1*a*). Later in the review we discuss the potential for future work to investigate how other niche‐based mechanisms such as competition and interspecific interactions influence community assembly (see Section IV).

#### (a) *Niche filtering along environmental gradients*

Niche filtering (or environmental filtering) occurs when the abiotic or biotic environment imposes barriers to establishment and/or survival, thus favouring the co‐occurrence of individuals with similar traits. It then follows that the typical signature of niche filtering is a non‐random pattern of clustering among trait values (functional clustering) in the emergent community (Weiher & Keddy, 1995a; Maire *et al*., 2012). However, other niche‐based mechanisms may also produce a similar pattern (Mayfield & Levine, 2010). Functional clustering may be revealed by comparing the observed dispersion in trait values – calculated from functional diversity metrics (reviewed in Mouchet *et al*., 2010) such as Rao's entropy and functional dispersion (FDis) – to random expectations from a null model (Villéger *et al*., 2008; Cadotte & Tucker, 2017). The effect of niche filtering may also be inferred from a shift in the community‐level weighted mean (CWM) – the mean of trait values in a community, weighted by the relative abundance of taxa bearing each trait value (Ricotta & Moretti, 2011). Below we summarize the effects of niche filtering observed along commonly studied environmental gradients, and highlight the response traits found to be indicative of the niche‐filtering process.

##### (i) *Climatic, altitudinal, and latitudinal gradients*

Given that thermoregulation is important for activity and survival in ectotherms (Heinrich, 1996), temperature is likely an important niche‐filtering mechanism for terrestrial arthropod communities. Initial findings from trait‐based studies examining climatic, altitudinal and latitudinal gradients generally support this notion. Especially among communities of social insects such as ants and bees, where thermoregulatory and thermophilic behaviours are widely documented (e.g. Stabentheiner & Kovac, 2014; Shi *et al*., 2015), a trait‐based approach has shown that niche filtering along temperature gradients (correlated with altitude) is driven by selection on physiological response traits measuring performance (survival), such as species' upper and lower thermal limits, which were higher in warmer environments and lower in colder environments, respectively (Peters *et al*., 2016; Bishop *et al*., 2017; but see Nowrouzi *et al*., 2018). The relationships between species' thermal tolerances and their altitudinal ranges were also employed to test predictions of the climatic variability hypothesis (Janzen, 1967), with contrasting results (see Bishop *et al*., 2017; Nowrouzi *et al*., 2018). Likewise, studies examining altitudinal and latitudinal patterns in the morphological and behavioural response traits of similar communities suggest that increased demands for thermoregulation in colder climates at higher altitudes or latitudes could explain the observed functional clustering of species with larger body sizes and increased pilosity (Bishop *et al*., 2016; Osorio‐Canadas *et al*., 2016; Peters *et al*., 2016; Costa *et al*., 2017), darker colour (Bishop *et al*., 2016), as well as ground‐nesting habits and higher sociality (Hoiss *et al*., 2012; Reymond *et al*., 2013; but see Purcell, 2011). Few studies have investigated the potential for gradients in aridity to structure communities, although Wiescher, Pearce‐Duvet & Feener (2012) observed that ant communities from environments of contrasting aridity did not differ significantly in desiccation resistance (but see Hood & Tschinkel, 1990). In general, trait‐based studies on terrestrial arthropod communities distributed along climatic, altitudinal and latitudinal gradients often identify body size as a response trait that is indicative of niche filtering; however the direction of the relationship varies both within and among taxonomic groups (e.g. Leingärtner, Krauss & Steffan‐Dewenter, 2014; Gibb *et al*., 2015; Osorio‐Canadas *et al*., 2016; Classen *et al*., 2017; Costa *et al*., 2017). Crucially, not all the above investigations of trait–environment relationships were necessarily theory‐driven (thus failing to reveal ecological mechanisms and limiting the predictive value of the results); the exception being body size–temperature relationships, which were often compared with the theoretical expectations of Bergmann's rule (Osorio‐Canadas *et al*., 2016; Peters *et al*., 2016; Costa *et al*., 2017). Notably, Classen *et al*. (2017) also tested whether intraspecific and interspecific variances in the body sizes of bees along altitudinal clines conformed to contrasting theoretical expectations of body size–temperature relationships under ‘physiological constraints hypotheses’ such as Bergmann's rule, and ‘energy constraints hypotheses’ which focus on how resources are allocated in size‐structured communities (Brown & Maurer, 1989). It should also be emphasized that environmental variation along climatic, altitudinal and latitudinal gradients is multidimensional (e.g. variation in temperature, aridity, habitat structure, resources). Hence, studies investigating the mechanisms driving coexistence along these gradients should not only be grounded in theory, but also aim to quantify multidimensional environmental variation, and prioritize the measurement of relevant performance traits (e.g. thermal and desiccation performance and/or tolerance). Notably, other studies have used community phylogenetics to show evidence for niche filtering with increasing elevation (e.g. Brehm, Strutzenberger & Fiedler, 2013; Smith, Hallwachs & Janzen, 2014). Unlike trait‐based approaches, however, such methods cannot yield information on the precise ecological mechanisms by which niche filtering occurs. Furthermore, studies using ‘phylogenetic‐patterns‐as‐proxy’ approaches tend to overlook the potential for evolution to have varying impacts on traits across the phylogeny, and as such are prone to accepting unsubstantiated assumptions (Gerhold *et al*., 2015; Cadotte, Davies & Peres‐Neto, 2017; but see Tucker *et al*., 2018). For example, phylogenetic niche conservatism is often implicitly assumed, although it may not be supported (Münkemüller *et al*., 2015).

##### (ii) *Gradients in habitat structure*

Several studies have measured the structural attributes of habitats such as vegetation height, aboveground plant biomass, canopy cover and landscape heterogeneity to investigate whether gradients in habitat structure could act as niche filters. Most focused on flying insects, under the assumption that habitat structure could potentially impact their dispersal and foraging niches, as well as others associated with flight (e.g. exposure to predators). Studies on European Lepidoptera report equivocal results. Some found evidence for functional clustering in homogenous habitats with shorter vegetation, where species with higher mobility, growth and fecundity were selected for (Aguirre‐Gutiérrez *et al*., 2017; Halder *et al*., 2017); however Hanspach *et al*. (2015) observed an opposite relationship where similar traits were selected for in more heterogeneous habitats, and Scalercio *et al*. (2012) did not observe significant effects of habitat structure on functional diversity. In a unique attempt to distinguish between community responses to compositional landscape heterogeneity (the diversity of habitat types) and configurational landscape heterogeneity (the number, size and arrangement of habitat patches), Perović *et al*. (2015) found that taxonomic diversity increased with compositional heterogeneity, but only high configurational heterogeneity selected for lepidopterans of larger body size and lower mobility. Habitat complexity has been suggested to impact the specific foraging niches of different arthropods. Complex habitats in tropical forests selected for larger bees that could potentially travel greater distances to locate trophic resources, but did not affect the functional structure of moth communities that may have had immediate access to abundant trophic resources (Costa *et al*., 2017). Similarly, reduced complexity (decreased ground cover) selected for ants possessing longer legs, which were conceivably advantageous for movement and resource discovery in simpler environments (Wiescher *et al*., 2012).

##### (iii) *Anthropogenic land‐use gradients*

There are many studies investigating whether increasing land‐use intensity functions as a niche‐filtering mechanism for terrestrial arthropod communities. Perović *et al*. (2018) recently reviewed some of these studies in detail from the perspective of landscape management; here our discussion focuses on broad trends. It appears that high intensities of land use in simplified, less‐heterogeneous habitats such as grasslands, pastures and farmlands can produce broad effects of niche filtering, creating functionally clustered communities with taxa of smaller body size, higher mobility or dispersal, and reduced ecological specialization (e.g. more generalist diets and nesting strategies). These effects have been observed across numerous taxa with diverse ecologies and spanning multiple trophic levels such as bees, beetles, butterflies, hemipterans, orthopterans, spiders (Börschig *et al*., 2013; Rader *et al*., 2014; Forrest *et al*., 2015; Gámez‐Virués *et al*., 2015; Mazzia *et al*., 2015; Simons, Weisser & Gossner, 2016; De Palma *et al*., 2017; Hanson *et al*., 2017; but see Perović *et al*., 2015; Le Provost *et al*., 2017; Ng *et al*., 2018), and soil‐dwelling arthropods in dozens of orders (Birkhofer *et al*., 2017; Rigal *et al*., 2018). By contrast, the effects of high‐intensity land use on the functional structures of arthropod communities may be buffered by landscape heterogeneity in relatively complex habitats such as forests (Edwards *et al*., 2014; Gossner *et al*., 2013; Gámez‐Virués *et al*., 2015; Perović *et al*., 2015; Birkhofer *et al*., 2017; Gómez‐Cifuentes *et al*., 2017; Murray *et al*., 2017; Salas‐Lopez *et al*., 2018; but see Martello *et al*., 2018). It is important to note that most studies were conducted in temperate regions; additional studies incorporating varying landscapes and land‐use practices (grazing, logging etc.) in the tropics are needed to explore the generality (or contingency) of land‐use intensity as a niche‐filtering mechanism for terrestrial arthropods. Global meta‐analyses on individual groups have made progress towards prediction by identifying response traits (e.g. bee nesting location and sociality; ant body size) that were broadly predictive of communities' functional responses to increasing land‐use intensity across different land‐use types and climates (Williams *et al*., 2010; Gibb *et al*., 2017; da Encarnação Coutinho, Garibaldi & Viana, 2018; but see Bartomeus *et al*., 2018). Finally, that functional diversity responded to land use in the absence of similar responses in taxonomic diversity (e.g. Forrest *et al*., 2015; De Palma *et al*., 2017) suggests that taxonomic approaches alone cannot fully account for anthropogenic impacts on biodiversity – this highlights the value of trait‐based approaches to research and conservation.

While the influence of niche filtering has been demonstrated in experimentally assembled plant communities (e.g. Weiher & Keddy, 1995b), the majority of trait‐based studies investigating niche filtering in terrestrial arthropod communities were observational. This is noteworthy because observational studies have limited potential for distinguishing the effects of niche filtering from those of other niche‐based mechanisms such as dispersal limitation and competition (Mayfield & Levine, 2010; HilleRisLambers *et al*., 2012). Still, observation‐based inferences of niche filtering may be strengthened if these are supported by three independent lines of evidence (Cadotte & Tucker, 2017). First, evidence of functional clustering should be demonstrated, such as by showing that the standardized effect sizes of trait distances, in relation to an appropriate null expectation, are significantly less than zero. Second, the functional clustering must be shown to be associated with an environmental gradient; this requires the measurement of environmental properties and statistically relating these to observed functional clustering. Finally, there should be evidence of a direct link between community structure and potential environmental drivers; this may be achieved by demonstrating that the environmental conditions where species are found, or where they attain maximal abundance, are non‐randomly related to species traits. A small number of terrestrial arthropod trait‐based studies investigating niche filtering do not provide robust evidence for the latter two criteria. In particular, environmental gradients were not measured; instead they were either assumed (e.g. assuming that temperature gradients directly correlate with altitudinal gradients) or qualitatively described (e.g. ‘low’, ‘medium’ and ‘high’ categories for land use). Consequently, efforts to demonstrate direct links between functional structure and environmental drivers were hindered. As the pitfalls of observational studies on niche filtering were only highlighted recently (Mayfield & Levine, 2010; Cadotte & Tucker, 2017), we foresee that future trait‐based studies on terrestrial arthropods will be designed better to address current limitations. For instance, novel approaches to improve the quantification of environmental gradients have begun to emerge, such as the measurement of functional diversity of the local plant community – a possible alternative to qualitative descriptors of resource gradients (Pellissier *et al*., 2013; Pakeman & Stockan, 2014).

#### (b) *Effects of disturbances on assembly and community structure*

Disturbances are abiotic or biotic forces or processes that result in perturbations, where ecosystems deviate – that is, are changed – from their reference states (Rykiel, 1985). Below we highlight trait‐based studies that have revealed how the assembly and functional structures of terrestrial arthropod communities are shaped by various abiotic and biotic disturbances, often in ways that would not be deciphered by taxonomic approaches alone.

##### (i) *Fire*

In line with theoretical expectations (Swengel, 2001; Schowalter, 2012), trait‐based studies have shown that fire generally shapes the assembly of terrestrial arthropod communities by causing local extinction of their original populations, and enabling rapid colonization by species that have high dispersal ability, which are able to tolerate the altered microclimate (e.g. open, less shady) and exploit the altered diversity of resources (e.g. food and reproduction substrates) in the post‐fire environment (Moretti *et al*., 2010; Heikkala *et al*., 2016). Positive effects of fire on functional diversity are reported for communities of ants, bees and saproxylic beetles (Moretti *et al*., 2010; Arnan *et al*., 2013; Lazarina *et al*., 2016; but see Heikkala *et al*., 2016). In ants and bees, fire functioned as a niche‐filtering mechanism, where species with ground‐nesting colonies and ecological plasticity (e.g. polymorphism, polylecty) were selected for in fire‐prone habitats (Arnan *et al*., 2013; Lazarina *et al*., 2016). By contrast, fire reshaped the functional structure of saproxylic beetle communities through contrasting mechanisms acting on different niches. While altered climatic conditions functioned as a niche‐filtering mechanism that selected for species with narrow climatic requirements (Moretti *et al*., 2010; Heikkala *et al*., 2016), the simultaneous release of diverse food sources in post‐fire conditions increased resource opportunities, which shifted average trait values and expanded trophic niche space (Moretti *et al*., 2010). Comparative studies have also demonstrated the contrasting effects of fire on the functional structures of bee communities in the Mediterranean (unchanged) and temperate regions (high functional replacement); here, an assessment of functional structure was crucial as the communities' responses in species diversity were similar (Moretti *et al*., 2009). In general, one might expect the effects of fire on the functional structure of arthropod communities to differ between biomes where it is frequent and natural (e.g. many grasslands) and those where it is relatively infrequent (e.g. tropical rainforests).

##### (ii) *Floods*

Like fire, flood events generally act as strong niche‐filtering mechanisms across multiple taxonomic groups (Dziock *et al*., 2011; Gerisch, 2011, 2014; Gerisch *et al*., 2012; Fournier *et al*., 2015); although it must be noted that as these studies were conducted in temperate floodplains, the relationships are not generalizable to other arthropod communities experiencing different flood pulses (e.g. Amazonian floodplains; Adis, 1997). While relationships vary among taxa, most of the above studies observed that small‐bodied and highly mobile (especially flying) species were selected for in areas experiencing regular floods, highlighting the importance of dispersal ability in the re‐colonization of previously flooded areas (Dziock *et al*., 2011; Gerisch *et al*., 2012; Fournier *et al*., 2015). Trait values promoting rapid population recovery after re‐colonization such as adult overwintering and high fecundity (ovariole number) were also selected for in some instances (Dziock *et al*., 2011; Gerisch *et al*., 2012). Trait‐based approaches have also been useful for revealing contrasts between communities' taxonomic and functional responses, where regular flooding was associated with highest taxonomic diversity, but lowest functional diversity (due to the strong filtering on species with similar traits mentioned above) (Gerisch *et al*., 2012). Few trait‐based studies have explored the interactive effects of regular and stochastic disturbances on the functional structures of terrestrial arthropod communities, but floodplains may represent model systems for such work. For instance, sampling during regular and stochastic flood events, Gerisch (2014) hypothesized that the high functional redundancy (proportion of species sharing similar functions) of ground beetle communities shaped by regular disturbances (i.e. many species with high propensities for dispersal and population recovery) would be a stabilizing force during a stochastic disturbance. Following a rare extreme flood event, these communities indeed recovered their original levels of functional diversity more quickly than less functionally redundant communities shaped by irregular flood regimes (Gerisch, 2014).

##### (iii) Biological invasions

Few studies have used trait‐based approaches to investigate the functional responses of terrestrial arthropod communities to biotic disturbances. Within the context of biological invasions, studies generally observed that invasive plants altered the functional structures of arthropod communities through effecting dietary and habitat shifts (Schirmel & Buchholz, 2013; Grass, Berens & Farwig, 2014; Gomes, Carvalho & Gomes, 2018). However, in contrast to the sweeping effects of abiotic disturbances such as fires and floods, which can wipe out existing arthropod assemblages and generate subsequent filtering of the new colonizers, the precise effects of plant invasions vary considerably with habitat, arthropod community, and ecological characteristics of the invader. For instance, moss invasions on coastal dunes selected for larger‐bodied spiders and beetles, and led to the loss of some species of phytophagous beetles and web‐building spiders dependent on native vegetation for food and habitat – yet this produced contrasting effects on the functional diversity of the two groups (Schirmel & Buchholz, 2013). Additionally, while invasions by exotic plants produced the typical effects of niche filtering on flower visitors – favouring smaller‐sized species and reducing functional diversity (Grass *et al*., 2014) – in other cases, exotic plant invasions actually reduced the strength of niche filtering. For instance, Gomes *et al*. (2018) observed that invasions by *Acacia longifolia* on dunes mitigated the otherwise extreme environmental conditions, resulting in more functionally diverse spider communities, as xerophilic specialists were replaced by generalists possessing a wider variety of traits. We are not aware of trait‐based studies investigating the functional responses of terrestrial arthropod communities to closely related invaders of the same trophic level, although some studies have attempted to address these questions with community phylogenetics (e.g. Lessard *et al*., 2009). Thus, there is much scope for using trait‐based approaches to test various invasion hypotheses (see MacDougall, Gilbert & Levine, 2009) and to advance current understanding of the mechanistic processes that underpin biological invasions – many of which involve terrestrial arthropods (Lowe *et al*., 2000).

### (2) Investigating biodiversity–ecosystem function relationships

Knowledge of the effects of biodiversity on ecosystem functions (BEF relationships) is integral to safeguarding Earth systems and human wellbeing (Hooper *et al*., 2005). The value of trait‐based approaches for revealing the mechanistic bases of BEF relationships and improving their prediction has long been acknowledged in plant studies (Díaz *et al*., 2004; Lavorel & Garnier, 2002). However, only recently have comparable trait‐based studies on terrestrial arthropods and their associated ecosystem functions begun to emerge (Fründ *et al*., 2013; Barnes *et al*., 2014; Gagic *et al*., 2015). Several hypotheses describe how community functional structure may influence ecosystem function; these can be tested by the extent to which different distance metrics of functional diversity predict ecosystem function. First, if functional differences among species are unimportant, then the overall numerical or biomass abundance of organisms in a community might better predict ecosystem function than any trait‐based measure (null hypothesis). Second, if a single trait value is strongly linked to an ecosystem function, then the abundance of this trait value in the community – the CWM – may best predict ecosystem function (functional identity or mass ratio hypothesis; Grime, 1998). Third, ecosystem functions may depend on the complementarity of different trait values in the community (functional complementarity hypothesis; Díaz & Cabido, 2001; Tilman *et al*., 2001); here, the condition of complementarity may be fulfilled solely by the presence of trait value combinations – predicted by functional richness (FRic) or functional dispersion (FDis), or it may also be dependent on the relative abundance of trait values in combination – predicted by functional evenness (FEve), functional divergence (FDiv) or weighted FDis (Gagic *et al*., 2015).

Terrestrial arthropods are mediators of numerous ecosystem functions (Yang & Gratton, 2014; Noriega *et al*., 2018), but only a minority of these have been investigated in trait‐based studies on BEF relationships (Table 1). Across the studies, each hypothesis linking biodiversity to ecosystem functioning found support at least once (Table 1), and in some cases ecosystem functions were best predicted by both functional identity and functional complementarity, which are not mutually exclusive (see Loreau & Hector, 2001). This lack of consensus among the studies is likely attributable to their inherent differences in several key aspects. First, the extent of phylogenetic relatedness among species in a community varies considerably; while some studies use closely related communities (e.g. ants; Retana, Arnan & Cerdá, 2015), others use distantly related communities (e.g. isopods and millipedes; Coulis *et al*., 2015). Second, the types of interactions that constitute the focal ecosystem functions are dissimilar; for instance, predation and herbivory involve trophic interactions, but seed burial and seed dispersal may involve non‐trophic interactions. Third, important methodological differences among studies stem from both the overall study design (e.g. experimental manipulations of functional diversity *versus* observational studies), as well as the specific techniques used (e.g. different ways of measuring the same ecosystem function). Spatial scale is another vital factor to consider and may explain interaction effects between functional identity and functional complementarity. For instance, research on plant communities suggests that functional identity better predicts BEF relationships at larger spatial scales, where niche filtering along environmental gradients leads to the clustering of trait values that in turn dominate communities and ecosystem functions (Grime, 1998; Lavorel & Garnier, 2002; Díaz *et al*., 2004; Laughlin, 2014), whereas the importance of trait differences and their combinations (functional complementarity) should increase at smaller spatial scales, where competitive interactions shape local diversity patterns and promote functional dispersion (Cadotte *et al*., 2011; Laughlin, 2014; Cadotte, 2017). Essentially, such arguments allude to the influence of community dynamics on ecosystem functions – a relatively unexplored area in terrestrial arthropod trait‐based studies of BEF relationships.

**Table 1:** Examples of trait‐based biodiversity–ecosystem function (BEF) studies on a variety of terrestrial arthropods and ecosystem functions, and support for four different hypotheses describing how community functional structure influences ecosystem function: organism abundance (Null), functional identity (FI), functional complementarity by presence of trait values only (FC), and functional complementarity by presence and abundance of trait values (FCa)

		BEF hypotheses supported				
Taxa	Ecosystem function(s)	Null	FI	FC	FCa	Reference
Ants	Resource exploitation			√		Retana *et al*. (2015)
Ants	Resource exploitation		√	√		Salas‐Lopez *et al*. (2017)
Bees*	Pollination			√		Fründ *et al*. (2013)
Bees	Pollination		√		√	Gagic *et al*. (2015)
Bees	Pollination			√		Garibaldi *et al*. (2015)
Bees	Pollination			√		Martins *et al*. (2015)
Beetles	Dung removal		√			Barnes *et al*. (2014)
Beetles	Dung removal, seed burial		√		√	Gagic *et al*. (2015)
Beetles	Seed predation		√		√	Gagic *et al*. (2015)
Beetles*	Seed dispersal, seed burial			√	√	Griffiths *et al*. (2015)
Grasshoppers	Herbivory		√		√	Moretti *et al*. (2013)
Grasshoppers	Herbivory			√		Deraison *et al*. (2015)
Isopods*	Decomposition		√		√	Bílá *et al*. (2014)
Isopods and millipedes*	Decomposition			√		Coulis *et al*. (2015)
Multi‐taxa (leaf litter invertebrates)	Energy fluxes	√				Barnes *et al*. (2016)
Spiders*	Plant primary production (through top‐down control of herbivory)		√			Schmitz (2009)
Spiders and beetles	Predation		√			Rusch *et al*. (2015)

* Indicates a study that experimentally manipulated functional diversity.

The response–effect framework (Lavorel & Garnier, 2002; Suding *et al*., 2008) is useful for conceptualizing the effects of community dynamics on ecosystem functions. Specifically, it aims to predict the effects of environmental change on ecosystem functions by explicitly identifying linkages between response traits that determine community responses to environmental changes, and effect traits that determine the effects of those changes on ecosystem functions. Surprisingly few terrestrial arthropod trait‐based studies have embraced this framework in its entirety; many studies investigated responses (examples in Section II.1), and others examined effects (Table 1), but studies attempting to identify linkages between responses and effects are currently scarce. However, preliminary findings suggest that the identification of these linkages can improve the prediction of terrestrial arthropod BEF relationships. For instance, Barnes *et al*. (2014) observed that the assembly of dung beetle communities along a restoration gradient was mediated through selection on the response traits of dispersal ability and body size; subsequently, the functional identity (CWM) of body size in these communities was predictive of the rate of dung removal. Many ecosystem functions, however, ultimately rely on interactions between organisms of different trophic levels (e.g. predation, herbivory) (Reiss *et al*., 2009). To improve predictions for such ecosystem functions, Lavorel *et al*. (2013) expanded the response–effect framework by incorporating a multi‐trophic perspective and interaction networks. Applying the new framework, Moretti *et al*. (2013) showed that ecosystem function (biomass production) was predicted by effect traits of both producers (plants) and consumers (grasshoppers), as well as the interactions between them; these effect traits were in turn related to response traits that were selected across an environmental gradient of land use. The new framework was also recently modified to address top‐down processes such as biological control (Perovíc *et al*., 2018). However, in spite of these significant conceptual advances, a shortage of information on effect traits and their relationships to ecosystem functions remains a fundamental challenge to understanding terrestrial arthropod BEF relationships (Moretti *et al*., 2013). Experimental studies manipulating functional diversity across multiple traits and tracing the corresponding impacts on ecosystem functions (e.g. Deraison *et al*., 2015) will likely be most effective at identifying effect traits and quantifying their effects. Along with more standardized measures of ecosystem functions, such basic gaps will need to be filled before the explanatory and predictive potential of conceptual frameworks (e.g. Suding *et al*., 2008; Lavorel *et al*., 2013) can be realized.

## III. HOW CAN TRAIT‐BASED STUDIES ADDRESS OUTSTANDING ASSUMPTIONS AND LIMITATIONS?

**Figure 2:**
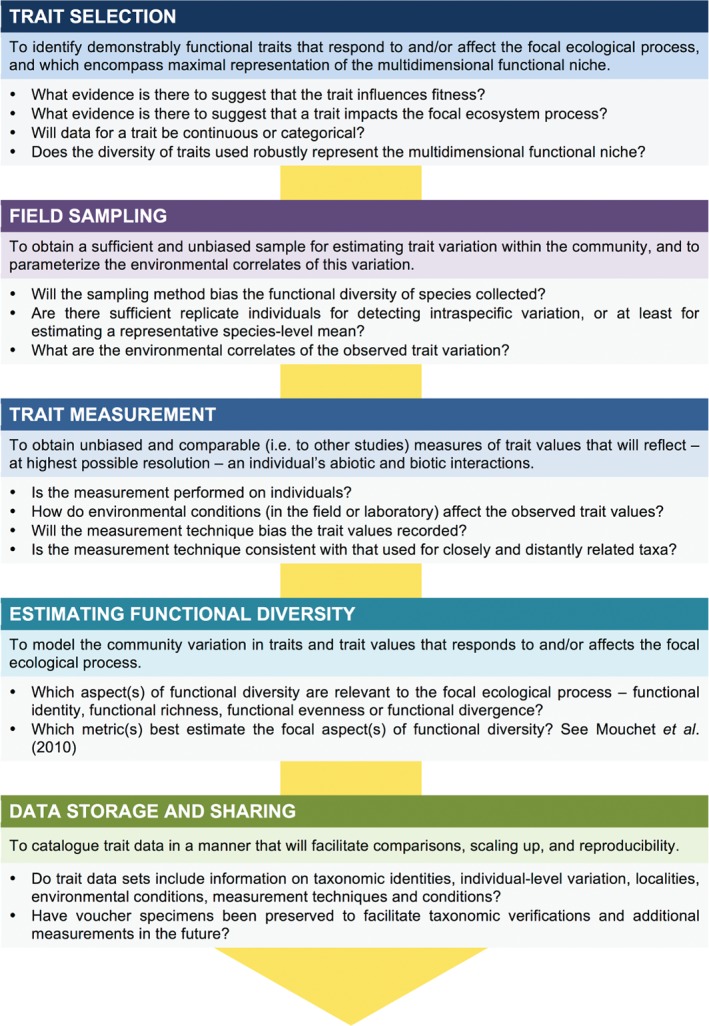
Roadmap for observational trait‐based studies on terrestrial arthropod communities. The majority of trait‐based studies on terrestrial arthropod communities rely on observational data. To provide accurate explanations for ecological phenomena and high‐quality trait data for further use, it is crucial that observational studies are robustly designed to address commonly overlooked assumptions and limitations. Here, objectives and relevant considerations for important stages of trait‐based research are suggested.

Here we highlight outstanding assumptions and limitations that presently impede research on terrestrial arthropods from realizing trait‐based ecology's ultimate promise of synthesis, generality and prediction (Shipley *et al*., 2016), and discuss how future research can address these issues. As the majority of trait‐based studies on terrestrial arthropods will likely remain observational, we also present guidelines for future work in this area (Fig. 2).

### (1) Assumption: functional traits are functional

A fundamental assumption of the trait‐based approach is trait functionality; all studies should use traits that possess fitness‐functionality, and where BEF studies are concerned, those traits should also possess ecosystem‐functionality. However, these assumptions of trait functionality have not been explicitly tested for the overwhelming majority of terrestrial arthropods, and the problem is further compounded by the diversity of traits recorded for each species. For instance, some studies used multiple morphological measurements with limited evidence for the functionality of these separate ‘traits’ (Wiescher *et al*., 2012; Mickaël *et al*., 2015). It is not uncommon for morphological traits to be included on the basis of their accessibility to measurement – but this is an incorrect approach. There is evidence to suggest that some traits do not predict growth, reproduction nor survival (Yang, Cao & Swenson, 2018); that some traits do not actually respond to or impact ecosystem processes (Mlambo, 2014; Bartomeus *et al*., 2018); and that trait expression can be flexible (Schmitz *et al*., 2015). Using traits when there is no evidence for their functionality runs the risk of attributing patterns in community functional structure to false mechanisms. The important task of establishing the fitness‐functionality of traits (the fundamental criterion) will be challenging because fitness‐functionality is essentially a complex dynamic outcome, influenced by the interaction of traits in multidimensional trait space as well as the environment. That is, within the individual organism, the contribution of one trait to fitness is influenced by the contributions of other traits to fitness, and the nature of these interactions (termed ‘trade‐offs’; Lavorel & Garnier, 2002) change along environmental gradients (Laughlin & Messier, 2015). Identifying predictable patterns (e.g. trade‐offs and correlations) along major axes of trait variation in multidimensional trait space can significantly enhance the process of trait selection if they allow for the use of a few, easily measurable traits to represent species' relative positions along different axes (Westoby *et al*., 2002), thereby achieving a more robust and efficient characterization of their multidimensional niches. General patterns of multidimensional trait variation are relatively well established in plants (e.g. the leaf‐economics spectrum; Wright *et al*., 2004), and similar attempts to identify such patterns in terrestrial arthropods emerged recently (Ellers *et al*., 2018).

#### (a) *Experiments to determine trait functionality*

To investigate the fitness‐functionality of terrestrial arthropod traits rigorously, assessments of trait–fitness relationships along environmental gradients are needed. For instance, by measuring the traits of 66 tree species grown in controlled conditions, Kramer‐Walter *et al*. (2016) identified trade‐offs among multidimensional root and leaf traits, and their corresponding impacts on fitness (i.e. growth) along a gradient of soil fertility. Similar experiments have been performed with well‐studied insect models, such as in *Drosophila serrata*, where thermal reaction norms for multiple morphological traits were assessed along a thermal gradient (Liefting, Hoffmann & Ellers, 2009). Such work can provide a framework for investigations on other terrestrial arthropods amenable to field and laboratory mesocosm experiments, where the direct effects of trait variation on fitness can be estimated by measuring performance in terms of growth, reproduction and survival. Here, it will be essential to select traits on theoretical bases of their functionality so that the results contribute to elucidating mechanisms. It will also be crucial that assessments of trait–fitness relationships are assessed along measured environmental gradients, and that a ‘trait × environment’ interaction term is factored in to explain fitness; leaving this out would imply that some set of trait values can confer fitness in all environments – an over‐simplistic and impossible scenario (Laughlin & Messier, 2015). In determining the fitness‐functionality of traits along environmental gradients, investigators may simultaneously adopt the response–effect framework (Lavorel & Garnier, 2002; Suding *et al*., 2008) to measure the ecosystem processes regulated by the organisms' performance along these gradients; this approach would be advantageous, as it would allow investigators to establish explicitly the ecosystem‐functionality of traits or trait sets in relation to fitness and the environment. Experimentally obtained information on the fitness‐ and ecosystem‐functionality of individual traits and trait sets, trends in trade‐offs and correlations, and the environmental correlates of trait interactions may also improve *a priori* trait selection in observational studies on similar taxa.

#### (b) *Trait functionality in observational research*

Naturally, experiments involving performance measures along environmental gradients can only be undertaken for a fraction of terrestrial arthropods. However, standards for observational trait‐based studies can still be raised so as to enhance their potential for synthesis, generality and prediction. First, investigators should strive to justify explicitly the functionality of the traits used. In the absence of evidence, traits should at the very least be selected on the theoretical bases of their functionality – that is, how their abiotic or biotic interactions may impact specific fitness components (growth, reproduction or survival) and relevant ecosystem processes. Selecting traits in this manner will better facilitate a mechanistic understanding of observed empirical patterns (i.e. trait–environment relationships) to generate good predictions. Essentially, only traits with demonstrable indirect or direct links to performance should be selected, while also balancing this with the important objective of maximizing the functional niche represented (achieved by sampling traits from different functional dimensions) (Laughlin, 2014; Kraft, Godoy & Levine, 2015). One approach for positing fitness‐functionality might be to establish links between traits from different levels of the performance hierarchy. If lower traits that are more accessible to measurement (e.g. pilosity, cuticle thickness) can predict interactions between the environment and higher traits (e.g. foraging activity) or performance (e.g. thermal tolerance, survival), then there may be some grounds for using the lower traits as proxies (within those environmental parameters). In ants, some morphological traits were found to be associated with trophic position, and these associations persisted after correcting for phylogeny (Gibb *et al*., 2015). Likewise, morphological traits were recently shown to predict nesting behaviours and activity periods in dung beetles (Raine *et al*., 2018). However in most cases morphological traits were used without evidence of any associations between morphological traits and higher traits, let alone their effects on performance. As previously mentioned, the ecosystem‐functionality of putative effect traits may be explored in pilot studies assessing how different values of these traits impact ecosystem functions (e.g. Deraison *et al*., 2015).

Second, investigators should quantify the environmental gradients that correspond to the observed variation in trait values. While many studies examined community functional structures across varying environments, only a minority linked this trait variation to a demonstrable (i.e. quantified) environmental gradient (e.g. Gerisch *et al*., 2012; Peters *et al*., 2016; Aguirre‐Gutiérrez *et al*., 2017). The inferred‐but‐unquantified environmental effects across many studies make the comparison of observed functional relationships impossible (hindering generality and synthesis). Such studies also have limited potential for predicting the responses of terrestrial arthropod communities to environmental changes, both in terms of their functional structure and their effects on ecosystem processes.

### (2) Assumption: effects of intraspecific variation can be ignored

Intraspecific variation in phenotypic traits within a species may be generated through mechanisms such as local adaptation, parental conditions, ontogeny and phenotypic plasticity (Des Roches *et al*., 2018). In studies on ecological relationships at, and above, the community level, the ecological consequences of intraspecific variation are often implicitly assumed to be negligible, or at least subservient to those of interspecific variation; most analyses are performed using only species‐level means (Violle *et al*., 2012; Didham *et al*., 2016). However, recent theoretical and empirical studies, spanning diverse taxa and multiple trophic levels, propose that because intraspecific effects are often comparable to, and sometimes stronger than, species effects (Siefert *et al*., 2015; Des Roches *et al*., 2018), they may influence species coexistence (Violle *et al*., 2012; Hart, Schreiber & Levine, 2016) and ecosystem functions (Johnson *et al*., 2012), and also determine ecological responses to global change (Moran, Hartig & Bell, 2016; Wright *et al*., 2016). Such notions have found support in trait‐based research on fungi (e.g. Hazard *et al*., 2017) and plants (e.g. Bennett *et al*., 2016), as well as animals (e.g. Ross *et al*., 2017) including terrestrial arthropods. Studies on wild bee communities have shown that intraspecific variation in body size may shape assembly along climatic gradients (Classen *et al*., 2017), and mediate the effects of habitat fragmentation on community structure and the ecosystem function of pollination (Warzecha *et al*., 2016). Similarly, intraspecific variation in the foraging and web‐building behaviours of several spiders has been shown to drive their community dynamics and responses to environmental change (Pruitt & Ferrari, 2011; Pruitt & Modlmeier, 2015; Dahirel *et al*., 2017), as well as changes in the structure of prey communities (Royauté & Pruitt, 2015).

The ecological consequences of intraspecific variation in most terrestrial arthropods remain unexplored (Didham *et al*., 2016). Yet trait‐based studies using only species‐level means will fail to detect the effects of intraspecific variation on community structure and dynamics (reviewed in Bolnick *et al*., 2011). Few studies have sought to investigate the ‘stable species hierarchy’ hypothesis, which predicts that trait variation is higher at the interspecific level than at the intraspecific level (but see Bonfanti *et al*., 2018). Intraspecific variation is reported to be high among particular traits of some arthropods [e.g. colouration in the ladybird beetle *Harmonia axyridis* (Koch, 2003); diet breadth of the fire ant *Solenopsis invicta* (Roeder & Kaspari, 2017)]; but low in others (e.g. morphology of dung beetles; Griffiths *et al*., 2016). Moving forward, investigators should explore the effects of intraspecific variation in terrestrial arthropods, such as by determining how individual differences in multidimensional trait space and in trade‐offs between traits influence performance along environmental gradients. Following this, the higher goal would then be to investigate how trade‐offs within species differ from trade‐offs among species, as this would improve understanding of functional trait evolution and the sorting of phenotypes across environmental gradients (Laughlin & Messier, 2015). To complement such work, which would likely be limited to experimental communities consisting of few species, the many observational studies investigating the assembly, dynamics and ecosystem functions of diverse species communities should move away from a total reliance on species‐level means, and instead strive to model the effects of intraspecific variation on community functional structure. For such purposes, a variety of analytical tools that compare intraspecific to interspecific trait variability (e.g. ‘T‐statistics’; Violle *et al*., 2012) and which incorporate intraspecific variation into the calculation of functional diversity metrics should prove useful (e.g. de Bello *et al*., 2011; Laughlin *et al*., 2012; Carmona *et al*., 2016). Here, a foreseeable challenge will be collecting and measuring sufficient individuals to provide a representative sample for each species. Previous studies measured from one to over 2000 individuals per species (Griffiths *et al*., 2016; Warzecha *et al*., 2016; Classen *et al*., 2017). Griffiths *et al*. (2016) recommend measuring ‘at least 30 individuals [per species] when working with invertebrate traits that are likely to display high levels of phenotypic plasticity’; however, they did not account for the effects of multidimensional trait covariances in their estimate. While 30 individuals per species may suffice when estimating a one‐dimensional normal trait distribution with 90% accuracy, at least 50 individuals per species are required to estimate a three‐dimensional trait distribution with 90% accuracy, and the minimum number of replicates increases with increasing trait dimensionality and with departure from normality (Laughlin & Messier, 2015). Such high demands for replicates underscore the importance of curating individual‐level trait information in multi‐species trait databases (discussed in Section III.4).

### (3) Limitation: sampling methods as trait filters

Most if not all sampling methods bias detection towards particular species and away from others. When sampling methods consistently fail to detect certain species because of their particular traits or trait values (detection filtering), the effects on observed trait–environment relationships may be substantial (Pakeman, 2014; Roth *et al*., 2018). Under‐sampling bias is especially pervasive for terrestrial arthropods, which are a difficult group to sample, and for which a wide variety of sampling methods have been developed (Coddington *et al*., 2009). However the potential effects of under‐sampling bias and detection filtering on the observed functional diversity of terrestrial arthropod assemblages are neither acknowledged nor addressed in most trait‐based studies. It is conceivable that detection filtering could operate on traits such as body size (e.g. pitfall traps are more effective at capturing larger‐sized ants; Olson, 1991), mobility (when fast‐moving species escape sweep nets), diet (if baits are used in collections), seasonality and activity time (when sampling is conducted during a specific season and time of day), and many others. Notably, these traits are commonly used in trait‐based studies on terrestrial arthropods. Moving forward, studies investigating the direction and strength of detection filtering by widely used sampling methods for terrestrial arthropods will contribute practical information to guide method selection in trait‐based work. One approach to reduce under‐sampling bias may be to incorporate a greater variety of sampling methods in the protocol (Bestelmeyer *et al*., 2000); however this may also impede efforts to standardize sampling across studies, which is crucial for the comparison of results and the integration of trait data. While it is seldom possible to remove all biases from the sampling process, it remains imperative that investigators recognise – and also report – how these may affect the trait–environment relationships and patterns of functional diversity observed.

### (4) Limitation: source, structure and consolidation of trait information

Information on traits may be obtained from the primary source – that is, through observation and measurement on organisms encountered during the study – or from secondary sources such as the literature and data repositories. The structure of trait data may be in continuous form (e.g. body size in mm, clutch size) or categorical form (e.g. colour, diet). The particular source and structure of trait information used will affect the quality of results obtained. Where possible, investigators should prioritize the collection of primary data on traits, and in continuous form. Primary data on traits measured *in situ* will be most representative of the ecological relationships investigated, since these are the traits directly undergoing selection by, or affecting, the studied environment. Continuous data will better reflect changes in the intensities of a trait's interactions than discrete categorical data (Lavorel & Garnier, 2002). Using primary and continuous data will also be important if potential effects of intraspecific variation are to be addressed. However, across the studies presently reviewed, the use of trait data from secondary sources and in categorical form was very common. As one example, among studies on lepidopterans, ‘larval diet breadth’ was often estimated *via* a ranked variable, which recorded whether the larvae of a species fed on ‘(*i*) one plant species; (*ii*) two plant species; and (*iii*) three plant species, etc.’ (Diamond *et al*., 2011, p. 1006), or alternatively ‘(*i*) one plant species; (*ii*) more than one species from the same genus; (*iii*) more than one genus from the same family; and (*iv*) more than one family from the same order, etc.’ (Graça *et al*., 2016, p. 302). This information was either obtained from the literature (e.g. Hardy *et al*., 2007) or databases (e.g. Janzen & Hallwachs, 2009); and where information was absent for a particular species, information from a congeneric was used in its place (Graça *et al*., 2016, 2017).

Of course, it will not always be feasible to collect primary data on traits. There is a general lack of knowledge on species' traits, ecological functions and interactions (see the ‘Raunkiearan’ and ‘Eltonian’ shortfalls; Hortal *et al*., 2015). Furthermore, acquiring trait information is especially difficult in studies investigating relationships at global and regional scales where primary sampling may be impractical, as well as studies on rare and endangered species where collecting specimens may be difficult or prohibited (although phylogeny‐based imputation may be one way to reconcile missing trait data; see Penone *et al*., 2014). In this regard, the value of consolidating high‐quality trait data in comprehensive and organized data repositories cannot be overstated. Several of these already exist for terrestrial arthropods such as ants (e.g. GLAD; Parr *et al*., 2017), ground beetles (e.g. http://carabids.org; Homburg *et al*., 2014) and soil invertebrates [BETSI (Hedde *et al*., 2012); Edaphobase (Burkhardt *et al*., 2014)]; there are likely to be many more databases that are unpublished. Previous authors have provided recommendations for managing the eco‐informatics of trait databases (Schneider *et al*., 2018), including those for terrestrial arthropods (Pey *et al*., 2014). Below we propose seven additional recommendations for the development of terrestrial arthropod trait databases, focusing on the collection of trait information.

First, maintaining accurate taxonomic identifications and updates for species' trait data is paramount, as these are the primary means for identifying and comparing trait information. This will be a significant challenge as substantial taxonomic inaccuracies are prevalent in most biological databases (Goodwin *et al*., 2015; Maldonado *et al*., 2015) – and especially so for the terrestrial arthropods, where research is hampered by many taxonomic impediments (discussed in Section I.3). Thus, while data on the traits of terrestrial arthropods could accumulate relatively easily, advances in taxonomic research will be important for effectively curating (including depositing and updating) these trait data in databases. One approach may be to tag trait data from the same individual with a DNA barcode or other genetic identifiers. Second, standardizing the measurement of traits is essential for facilitating comparisons among studies from different regions and localities; this potential for generality is a primary merit of the trait‐based approach (Moretti *et al*., 2017). Inconsistencies in trait information will be compounded when data from studies using different sampling methodologies and measurement techniques are compiled (e.g. in macroecological research). Third, prioritizing continuous data over discrete categorical trait data will improve the resolution of trait information and its potential to reflect intensities in trait interactions (Lavorel & Garnier, 2002). Fourth, prioritizing information on individual‐level trait variation will facilitate much‐needed research on intraspecific trait variation. This may be especially important for species with wide geographic distributions. Fifth, depositing information on sampling and measurement methodology, as well as the environmental correlates of observed trait values will be important for clarifying inconsistencies and also enhancing potential for reproducibility. Sixth, even if specimens used in ecological studies are not taxonomically identified, storing vouchers – and also their linked genetic sequence data, if possible – will facilitate the verification of trait information and reproducibility in the long term (Turney *et al*., 2015; Packer *et al*., 2018). Finally, incorporating trait data from digital and physical collections of museums, which are vast, will be especially useful for temporal and spatial comparisons.

## IV. NEW FRONTIERS FOR TERRESTRIAL ARTHROPOD TRAIT‐BASED ECOLOGY

In this section we highlight several areas that represent promising avenues for future trait‐based research on terrestrial arthropods. The research proposed here is especially well suited to investigations in terrestrial arthropod systems, likely to advance understanding of broad ecological phenomena, and should also enhance real‐world practice in ecological management and conservation.

### (1) Idiosyncratic traits of terrestrial arthropods

The sheer biological diversity of terrestrial arthropods affords new and exciting opportunities for exploring the ecological effects of distinct phenotypes that are absent from other organisms. Many of these idiosyncratic traits have not been investigated in trait‐based studies thus far. Especially relevant to intraspecific variation are traits that reflect ‘personality’: temporally consistent individual differences in behaviour along one or more behavioural axes (Modlmeier *et al*., 2015). Several studies on predator–prey and pollination networks suggest that variation in personality traits relating to foraging, resource use, and responses to predators can impact community structure and/or ecosystem functions through interspecific interactions (Hawlena, Hughes & Schmitz, 2011; Pruitt & Ferrari, 2011; Pruitt & Modlmeier, 2015; Royauté & Pruitt, 2015; Venjakob *et al*., 2016). However there remain numerous arthropod species varying in personalities and behavioural repertoires (e.g. circadian activity, territoriality, sexual interactions, sociality) – for which the effects on community structure, dynamics and ecosystem functions remain unexplored. Extended phenotypes may also potentially be considered functional traits if they influence interactions and individual fitness (Violle *et al*., 2014); for instance, in plants, phyllosphere bacterial diversity plays a key role in plant functioning (Kembel *et al*., 2014). How the diversity of extended phenotypes among terrestrial arthropods – such as the structural diversity of galls of gall‐making insects, webs of spiders, and nests of social insects – relates to their individual fitness, and interacts with the environment is relatively unexamined (but see Stone & Cook, 1998; Dahirel *et al*., 2017). Given the substantial biomass of these taxa in many environments, trait‐based research focusing on extended phenotypes may be the key to understanding a variety of important ecosystem functions (e.g. predation by spiders, biogeochemical processes of termite and ant nests). Other interesting questions may be explored by examining chemical traits [e.g. how do different defensive chemical compounds influence survival among communities of herbivorous insects? (see Zvereva & Kozlov, 2016)] as well as traits across life stages (e.g., in holometabolous insects, are the traits of larval and pupal stages indicative of niches and fitness of adults?). Importantly, future research in any of these areas should not assume but rather test explicitly the functionality of the focal traits; this will facilitate the scaling of trait interactions from individuals to ecosystems.

### (2) Competition and coexistence

The biotic force of competition has traditionally been thought to shape community assembly by preventing individuals with very similar niches from coexisting (MacArthur & Levins, 1967). Consequently, it has been posited that the intensity of competition decreases as two species diverge in trait values (trait dissimilarity) (MacArthur & Levins, 1967), and that competition produces a non‐random pattern of dispersion among trait values (functional overdispersion) in the community (Weiher & Keddy, 1995a; Maire *et al*., 2012). Accordingly, a few studies on ants, bees and spider communities have cited patterns of trait dissimilarities or functional overdispersion – mainly in feeding traits – as evidence for the influence of competition in assembly (Houadria *et al*., 2015; Michalko & Pekár, 2015; Litchenberg, Mendenhall & Brosi, 2017).

However, modern coexistence theory (Chesson, 2000; HilleRisLambers *et al*., 2012; Barabás, D'Andrea & Stump, 2018) proposes that relationships between competition and coexistence are more complex, and not solely dependent on species' dissimilarities in their niches (and traits). Rather, coexistence occurs when invasion growth rates are positive, resulting from a balance between the effects of stabilizing mechanisms (stabilization, *A*) (e.g. storage effects and relative non‐linearities) mediated by species' niche differences, and the effects of differences in competitive abilities (competitive advantages, *f*_*i*_) that favour particular species over others in the absence of stabilization (Barabás *et al*., 2018). In line with this theory, experiments on plant communities show that competition may actually result in functional clustering among community members if the traits in question are associated with competitive dominance (Narwani *et al*., 2013; Godoy, Kraft & Levine, 2014), and that particular trait values may confer competitive advantages independently from trait dissimilarity (Mayfield & Levine, 2010; Kraft *et al*., 2015). At the global scale, traits that consistently influenced competitive interactions in plant communities were also identified (Kunstler *et al*., 2016).

Integrating modern coexistence theory with trait‐based ecology may provide a powerful paradigm for tackling the fundamental question of coexistence (Kraft *et al*., 2015). However most empirical applications have been constrained to plant research. Trait‐based studies on terrestrial arthropods by and large have not investigated their community ecology from the perspective of modern coexistence theory. Often, traits which may conceivably contribute to *f*_*i*_ (e.g. body size, aggression) as well as performance traits which may conceivably directly impact growth rates (e.g. fecundity) are not distinguished from traits that are assumed to reflect niche differences, which potentially contribute to *A*.

Future trait‐based studies investigating the relative magnitudes and roles of *A* and *f*_*i*_ in determining coexistence in terrestrial arthropod communities may advance understanding of how their diversity is shaped and maintained. In addition, the effects of competition and coexistence on ecosystem functions may also be explored. For instance, how do ecosystem functions vary among communities that are robust (small *f*_*i*_, large *A*), dynamic (large *f*_*i*_, large *A*), unstable (large *f*_*i*_, small *A*) and quasi‐neutral (small *f*_*i*_, small *A*) (Adler, HilleRisLambers & Levine, 2007; Mayfield & Levine, 2010)? Such questions will have direct implications for the management of biodiversity and ecosystem services under changing environments. However, there are at least two obstacles and one caveat to the application of modern coexistence theory to empirical studies on terrestrial arthropods. The first obstacle will be identifying an appropriate spatial scale – one at which competition could potentially occur. Most resources that terrestrial arthropods compete for (e.g. plants, prey, nest sites) vary locally and across fine spatial scales, hence the many studies examining variation in community structure across broad environmental gradients (e.g. altitude) are not suited for detecting the influence of competition in assembly (Swenson *et al*., 2007). The second obstacle will be identifying the traits (or trait sets) that contribute to *A* and *f*_*i*_ in terrestrial arthropod communities, which are largely unknown at present. Elucidating these traits may require experiments that assemble communities of species at varying densities, and measuring multiple traits as well as growth rates for each species in each community (e.g. Kraft *et al*., 2015). Finally, as a caveat, modern coexistence theory may fail to explain coexistence in more diverse and complex arthropod communities because the theory assumes the absence of complex dynamics, the stability of the resident community, and the presence of only a few limiting factors – or at least fewer limiting factors than there are species in the community (see Barabás *et al*., 2018).

### (3) Structure and function across trophic levels in ecological communities

Competition among species within a trophic level does not always account for species coexistence at this level; nor does it explain the biodiversity of the ecological community, defined as all organisms living in the specified place and time (*sensu* Vellend, 2016). To do so requires understanding the wider array of non‐competitive interspecific interactions (including mutualism, facilitation, predation and parasitism), which may occur both within and across trophic levels (Levine *et al*., 2017). How these manifold interactions shape community dynamics and structure is poorly understood (Godoy *et al*., 2018). Terrestrial arthropods, which encompass diverse trophic levels and ecological strategies, are excellent systems for investigating such questions. Recent studies have successfully employed trait‐based approaches to elucidate the mechanistic bases of non‐competitive interactions, as well as their effects on community structure. For instance, a focus on traits revealed a strong role for Müllerian mimicry (in wing patterns) in shaping coexistence of butterfly species along altitudinal gradients (Chazot *et al*., 2014); while in a non‐trophic facilitative interaction, the functional diversity of cavity‐producing wood‐boring beetles influenced the body sizes of cavity‐nesting bees (Sydenham *et al*., 2016). Similarly, the functional diversity of socially parasitic rove beetles was shown to be driven by the abundance of their ant hosts (Psomas, Holdsworth & Eggerton, 2018). Recently, the identification of traits mediating trophic linkages between adjacent trophic levels (trait‐matching) has been useful for characterizing the structures of predator–prey (Brousseau, Gravel & Handa, 2018b), plant–herbivore (Le Provost *et al*., 2017) and pollination (Garibaldi *et al*., 2015; Bartomeus *et al*., 2016) networks. Identifying trophic linkages *via* trait‐matching also has facilitated assessments of the impact of environmental changes on multiple trophic levels (e.g. impact of landscape simplification on plant and herbivore functional diversity; Le Provost *et al*., 2017), as well as predictions on BEF relationships (e.g. in pollination networks, predictions of fruit set were enhanced by matching the body sizes and mouthpart lengths of flower visitors with the nectar accessibility of flowers; Garibaldi *et al*., 2015).

Moving forward, terrestrial arthropod trait‐based studies may take advantage of recent theoretical and conceptual advances to predict structure and function across multiple trophic levels. For instance, conceptual models using traits as the basic properties to integrate the structural components of ecological network research (e.g. trait‐matching and modular trophic units for interacting species) with the functional components of BEF research (e.g. species' responses to and effects on their environments) have been proposed to improve prediction of the many ecosystem functions encompassing multi‐trophic interactions (Schleuning, Fründ & García, 2015; Schmitz *et al*., 2015). Recently, Godoy *et al*. (2018) also presented a theoretical framework integrating niche and network theories to explain coexistence at multiple trophic levels, which explicitly considers the effects of stabilizing mechanisms as well as differences in competitive abilities. Future terrestrial arthropod trait‐based studies adopting these integrative frameworks may make important advances in explaining and predicting the emergent properties (structure and function) of complex ecological communities, and thus produce more accurate models of natural systems. Additionally, such research may also provide new insights into the BEF relationships of interspecific interactions such as mutualism (Schleuning *et al*., 2015) and parasitism (Frainer *et al*., 2018).

### (4) Functional biogeography of terrestrial arthropods

The emerging field of functional biogeography investigates the patterns, causes, and consequences of the geographic distribution of trait diversity (Violle *et al*., 2014). Trait‐based approaches are integral to this new field, which aims to (*i*) describe the distribution of traits along environmental gradients and across spatial scales; then, using this information; (*ii*) explain the geographic distribution of organisms, biodiversity patterns, and ecosystem processes; and (*iii*) predict responses to environmental changes using trait‐based predictive functions and models (Violle *et al*., 2014). Some of these aims have already been realized through the rapid progress of trait‐based plant ecology; for instance, high‐resolution world maps of plant trait variation were recently produced (Butler *et al*., 2017). The prospects for a functional biogeography of terrestrial arthropods are likewise promising. Applying trait‐based approaches to test hypotheses from the theory of island biogeography, Whittaker *et al*. (2014) showed that functional diversity–area relationships for spider and beetle communities scaled in a manner similar to species richness–area relationships across local, island and archipelagic scales. Basic questions of how terrestrial arthropod functional diversity and ecosystem functions scale with environment and area are presently unaddressed, and will likely be a core focus of future trait‐based research. Functional biogeography may also complement community ecology (and *vice versa*), by providing insights into the spatial scaling of assembly processes (e.g. species interactions) across broad gradients (Violle *et al*., 2014). We envisage that developing a functional biogeography of terrestrial arthropods will first entail extensive efforts to model trait–environment relationships at local and regional scales; importantly, these studies need to be comparable, and address current limitations (see Section III). This foundational work may then be integrated with species distribution models (e.g. GABI; Guénard *et al*., 2017), trait databases (e.g. GLAD; Parr *et al*., 2017) and statistical tools for scaling functional diversity (Carmona *et al*., 2016) to describe, explain and predict geographical distributions of terrestrial arthropod form and function. Finally, novel studies on trait–environment relationships across both space and time may usher in an exciting new field: ‘functional historical biogeography’ (Sukumaran & Knowles, 2018). For instance, might the geographic distributions and the traits of fossilized individuals, populations and communities reveal their relationships with prehistoric environments?

## V. CONCLUSIONS

1. By focussing on the functional properties of individual organisms, trait‐based ecology provides a broad, mechanistic framework for synthesizing, explaining and predicting structure and function across different levels of biological organization.
2. The time is ripe for a trait‐based ecology of terrestrial arthropods. Such work will improve understanding of the processes underlying patterns of diversity for complex ecological communities encompassing multiple trophic levels, and provide mechanistic insights to the functioning of essential ecosystem services such as pollination, biological control and nutrient cycling. For many terrestrial arthropods, taxonomic impediments have limited ecological research, and a focus on traits will be particularly useful for expediting understanding some of the interactions and functions of these taxa. The growing volume of data on the traits of terrestrial arthropods in databases and the wider literature, as well as physical and digital collections, will facilitate work on the generality of functional relationships across geographic regions and spatial scales.
3. Findings from pioneering studies on the trait‐based ecology of terrestrial arthropods attest to the value of this burgeoning field. Here, trait‐based approaches have been especially useful for elucidating the specific mechanisms driving the deterministic assembly of diverse communities across different environmental gradients, as well as their responses to disturbances – often revealing distinct patterns in functional diversity not detected by taxonomic and phylogenetic approaches. Preliminary work investigating terrestrial arthropod‐mediated ecosystem functions did not observe a consistent relationship between functional diversity and ecosystem function, although comparisons are strictly limited by differences among studies in terms of the focal scale, community characteristics and methods used.
4. New studies expanding the scope of terrestrial arthropod trait‐based research will advance knowledge in longstanding as well as emerging areas in ecology. As previous studies mainly focused on the interactions between traits and abiotic environments, one immediate avenue for future work will be to explore how biotic interactions – including various competitive, non‐competitive, and multi‐trophic interactions among species – shape structure and function across different environmental gradients and spatial scales. At the same time, novel research on the functional biogeography of terrestrial arthropods may succeed in mapping global distributions of their functional diversity, thereby enhancing ecological forecasting and the management of ecosystem services in the face of climate change and the spread of invasive species. Importantly, such efforts will be undermined if trait functionality is not rigorously tested, if the effects of intraspecific variation are not accounted for, and if the collection of trait information is biased or inconsistent. The predictive value of future work also rests upon the willingness of researchers to go beyond describing patterns to identify mechanisms – that is, by undertaking hypotheses‐driven investigations grounded in ecological theory. Future studies in terrestrial arthropod trait‐based ecology should thus explicitly address these aspects in the critical stages of study design, trait selection, sampling and measurement, as well as in the treatment and consolidation of trait data.
5. Realizing trait‐based ecology's higher goal of synthesis, generality and prediction also demands taxonomic excellence – to safeguard the accuracy and coherence of trait data. Thus it is important to recognize that functional approaches to describing biodiversity are ultimately complements, and not substitutes to taxonomic ones. While a focus on traits can help to overcome significant taxonomic impediments to the understanding of ecology, trait‐based research faces an even greater impediment – the lack of information on the form and functionality of organisms. Especially in the hyper‐diverse terrestrial arthropods, basic data are lacking on diet, physiology, phenology and behaviour – let alone information on how these vary intraspecifically along environmental gradients. In conclusion, we propose that reconciling contemporary trait‐based research with the long‐established study of taxonomy and natural history will pave the way for a more robust understanding of the mechanisms structuring arthropod diversity across space and time.
